# Can an Infectious Disease Genomics Project Predict and Prevent the Next Pandemic?

**DOI:** 10.1371/journal.pbio.1000219

**Published:** 2009-10-26

**Authors:** Rajesh Gupta, Mark H. Michalski, Frank R. Rijsberman

**Affiliations:** Google.org, Mountain View, California, United States of America

## Abstract

Infectious diseases need a globally coordinated genomic-based movement linking sequencing efforts to development of response tools to mitigate the impact of existing and emerging threats.

## Author Summary

The world of genomics is transforming medicine, and is likely to influence the future development of new drugs, diagnostics, and vaccines. To date, the greater focus of genomics and medicine has been on conditions affecting resourcewealthy settings, primarily involving scientists and companies in those settings. However, we believe that it is possible to expand genomics into a more global technology that can also focus on diseases of resource-limited settings. This goal can be achieved if genomics is made a global priority. We feel one way to move in this direction is through a comprehensive approach to infectious diseases—i.e., an Infectious Disease Genomics Project—that would mirror the Human Genome Project. Without an active, unified effort specifically focused on allowing actors at any level to participate in the genomics revolution, infectious diseases that primarily affect the poor will likely not achieve the same level of scientifici advancement as diseases affecting the wealthy.

We believe that there is great potential in the systematic application of genomics, proteomics, and bioinformatics to infectious diseases, and that this potential has yet to be fully realized. We suggest that the international community unite under an Infectious Disease Genomics Project, analogous to the Human Genome Project, with a goal of a comprehensive, open-access system of genomic information to accelerate scientific understanding and product development in the very settings where diseases have the highest probability of emerging. If properly structured, such an approach could shift fundamentally the global response to emerging infectious diseases.

## Genomics Is Systematically Transforming Medicine

The “Genomic Revolution” has transformed our vision and understanding of how living organisms and systems interact with each other and with the environment [1]. Increasingly, the science of genomics serves as the foundation for translational research for advancing the management of many important diseases [2]–[7]. Decreasing costs and increasing throughput of new technologies has made possible multinational collaboration on large-scale projects such as the Human Microbiome Project and the 1000 Genomes Project [8]–[10]. Infectious disease management is also transforming thanks to molecular technologies as seen in HIV [11],[12], tuberculosis [13],[14], malaria [15],[16], and other neglected tropical diseases [17],[18]. Discovering novel pathogens and elucidating the implications of genetic variation among existing pathogens [19],[20] is critical for rapidly mitigating pandemic threats, as demonstrated recently with severe acute respiratory syndrome (SARS) [21],[22] and avian (H5N1) and pandemic H1N1 2009 influenza (commonly referred to as “swine flu”) [23]–[26].

To fully harness the benefit of genomics in infectious diseases, a chain of overarching activities must occur. First, understanding the dynamics of infectious diseases through the genomics lens requires a tremendous amount of integrated comparative sequence, expression, epigenetic, and proteomic data from a variety of pathogens (bacteria, virus, protozoa, fungi), vectors (arthropod and avian sources), reservoirs (non-human mammals, environment) and human hosts. Second, generating, collating, organizing, and curating these data is an essential public health task. Third, translating this information to tools to improve surveillance and response mechanisms is critical to effectively impact disease management.

If this bench-to-beside chain of activities were optimized, we envision that the following could occur:

- Fully annotated genomes of all known pathogens, vectors, non-human hosts, and reservoir species, as well as a large number of candidate microbes in families that have a high risk of generating future pathogens, are held in public open-access databases such as GenBank.
- A “Genomic search” of all available contextual information, from sample origins through to published analyses, is as simple as a Google search.
- Sequencing and other molecular technologies are everyday tools-of-the-trade in every district hospital and laboratory in hotspots of emerging infectious disease, such as southeast Asia and sub-Saharan Africa.
- Automated molecular diagnostic assays are low-cost, reduced at least to the size of a smart mobile phone, and can return definitive diagnoses of a range of specialized known pathogen panels at the point of care.
- A range of products that use infectious disease genomic information routinely—such as vector maps, early warning systems, diagnostics, vaccines, and drugs—contribute to the prediction and prevention of epidemics.

While progress is occurring in each of these areas, the outputs—which are needed today—are far from complete.

## Creating an Infectious Disease Genomics Project (IDGP)

We believe that accelerated advances in the area of infectious diseases can occur under a global collaborative framework composed of discrete and delineated activities between the public and private sectors among resource-wealthy and resource-limited settings. The Human Genome Project (HGP) was a pioneering international effort that helped unlock the power of genomics for human health [27],[28]. This effort generated important information in part by having clear, targeted outcomes and by implementing a standard methodology across all participants. The HGP was a great impetus for progress seen thus far in genomics and health. Moreover, the HGP recognized that sequencing was just the first step in a much bigger process [26]. A similar effort for infectious diseases could, in our view, help predict and prevent the next pandemic.

**Figure 1:**
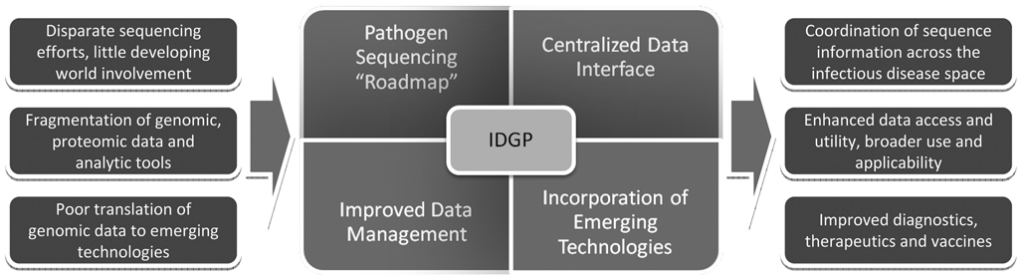
A coordinated Infectious Disease Genome Project (IDGP) could unify sequencing efforts, enhance data usability, and lead to essential tools for infectious disease management.

To capitalize on existing successful efforts in the area of genomics and infectious diseases such as those by the Broad Institute, Genomics Standards Consortium, J Craig Venter Institute, the National Institute of Allergy and Infectious Diseases, and the Wellcome Trust Sanger Institute (to name a few), we urge the international community to unite its numerous activities under an Infectious Diseases Genomic Project (IDGP)—a coordinated, large-scale, international effort focused on the genomes of pathogens, vectors, hosts, and reservoirs and linked to end-point surveillance and response systems. Such a project could coordinate activities in four specific areas: generating data, linking data, analyzing data, and applying data (Figure 1).

### Generating Data

At the outset, the IDGP would need to determine what the world requires in terms of genomic information. A standard approach to generating depth and diversity in genomic data is essential; beyond this, continuous real-time surveillance and characterization of evolving pathogens can help effectively forestall future epidemics/pandemics. Frontline work by consortiums, genome research centers, and individual laboratories has yielded baseline approaches in this area and a wealth of critical genomic information for many important infectious agents [29]–[34]. While each actor in the genomics field brings its own priority for targeting particular pathogens or diseases, a clear roadmap to generating a complete genomic picture of *all* infectious agents, emerging threats, hosts, and reservoirs, incorporating a broad range of investigators with varied technological capacity, would enhance both data generation and application. Such a process allows for community-level priority setting, thereby enabling smaller-scale laboratories to tailor projects to fit the needs of local communities while contributing to global efforts.

### Linking Data

The data collected must be connected to all relevant information and analytical tools in a single, easy-to-use, open-source, real-time interface. Such a system would improve on current systems by: gathering data across the public domain and working with companies/institutions to harness information in the private domain; linking accurate, annotated sequencing information to functional genomic and proteomic/functional proteomic information; attaching scientific literature associated with all levels of information; and including a self-sustaining financial mechanism potentially based on royalties from commercial products generated from the use of this system.

### Analyzing Data

The data need to be linked via large-scale, dynamic databases held in virtual servers allowing for collaboration and sharing while maintaining originating information for data rights and sovereignty. Concurrently, these data should be associated with a centralized collection of open-source bioinformatics tools capable of real-time operation in low- and high-speed computers and varying levels of internet connectivity. A single interface also would bring various sample collections together in formally structured biobanks that capture geospatial and context data to allow efficient scientific collaboration to take place. Centralizing the entire spectrum of information and analytic tools also allows researchers in resource-limited settings to participate in the genomics revolution without prohibitively costly machines, laboratories, and sample accessibility. Although we fully acknowledge that internet connectivity is a requirement that is not currently available to all, rapid technical innovation and investment from cheap netbook computers to new fiber optic cables in Africa are changing that equation. This system could be facilitated by virtual community collaboration or crowd-sourcing, taking full advantage of networking tools such as Wikipedia, Facebook, Twitter, FusionTables, and PLoS.

### Applying Data

Technological advances for basic scientific discovery (such as next-generation sequencers, microarrays, mass spectrometers, cell-based assay methods, and other tools for transcriptome, metabolome, and proteome discovery), novel techniques to increase throughput and/or decrease the cost of analysis, and applied clinical decision-making and surveillance tools (point-of-care diagnostics, rapid multi-pathogen assays) are in progress and should be supported actively. The IDGP should be informed by and incorporate emerging technology platforms to rapidly develop more accurate field diagnostics and to identify new opportunities for vaccine and drug development.

## Moving beyond Discourse into Action

An IDGP is attainable if others share this vision, show leadership, and see the added value resulting from a coordinated effort. The HGP certainly was a more targeted effort and we acknowledge that an IDGP will have additional obstacles to overcome. Scientific disagreement over targets is bound to occur. Complications resulting from the proposed level of data sharing should not be underestimated, and care must be taken to ensure proprietary rights and acknowledgement when warranted. Adapting molecular genetic technologies to resource-limited settings is a significant challenge, but is occurring with some success. Bringing together a community of scientists and donors, each with their own objectives and goals, to work under a single framework, is a difficult proposition. Finally, there will be many who will find this perspective simply too grandiose. Leaps of progress also require big visions, however, and it may just be possible that the 2009 H1N1 influenza pandemic is a enough of a reminder of what is at stake to provide a catalyst for action.

Google.org has supported global public health through its “Predict and Prevent” initiative with the aim of using the power of information and technology to address emerging infectious diseases by helping the world to know where to look for these diseases, find the threats earlier, and respond to them faster [35]. Google.org has focused its support on sequencing and pathogen discovery activities, bringing genomic technologies to resource-limited settings in East Africa, improving surveillance networks and systems, and exploring how our core competence in internet search can assist the infectious diseases community [36].

As firm supporters of the open access model for scientific publication [37], Google.org is pleased to support this series of essays, The Genomics of Emerging Infectious Disease, in partnership with the Public Library of Science (PLoS) journals (*PLoS Biology*, *PLoS Computational Biology*, *PLoS Genetics*, *PLoS Medicine*, *PLoS Neglected Tropical Diseases*, and *PLoS Pathogens*), not only to help define the current state of the art in pathogen genomics, but also, we hope, to stimulate debate on priorities for research and technology development.
